# New insights into quetiapine metabolism using molecular networking

**DOI:** 10.1038/s41598-020-77106-x

**Published:** 2020-11-16

**Authors:** Brendan Le Daré, Pierre-Jean Ferron, Pierre-Marie Allard, Bruno Clément, Isabelle Morel, Thomas Gicquel

**Affiliations:** 1 INSERM, INRAE, CHU Rennes, Institut NuMeCan (Nutrition, Metabolism and Cancer), PREVITOX Network, Univ Rennes, 35033 Rennes, France; 2 Forensic Toxicology Laboratory, Rennes University Hospital, 35033 Rennes, France; 3 School of Pharmaceutical Sciences, and Institute of Pharmaceutical Sciences of Western Switzerland (ISPSW), University of Geneva, CMU, Rue Michel Servet 1, 1211 Geneva 4, Switzerland

**Keywords:** Biological techniques, Cell biology, Biomarkers, Medical research

## Abstract

Metabolism is involved in both pharmacology and toxicology of most xenobiotics including drugs. Yet, visualization tools facilitating metabolism exploration are still underused, despite the availibility of pertinent bioinformatics solutions. Since molecular networking appears as a suitable tool to explore structurally related molecules, we aimed to investigate its interest in in vitro metabolism exploration. Quetiapine, a widely prescribed antipsychotic drug, undergoes well-described extensive metabolism, and is therefore an ideal candidate for such a proof of concept. Quetiapine was incubated in metabolically competent human liver cell models (HepaRG) for different times (0 h, 3 h, 8 h, 24 h) with or without cytochrom P450 (CYP) inhibitor (ketoconazole as CYP3A4/5 inhibitor and quinidine as CYP2D6 inhibitor), in order to study its metabolism kinetic and pathways. HepaRG culture supernatants were analyzed on an ultra-high performance liquid chromatography coupled with tandem mass spectrometry (LC-HRMS/MS). Molecular networking approach on LC-HRMS/MS data allowed to quickly visualize the quetiapine metabolism kinetics and determine the major metabolic pathways (CYP3A4/5 and/or CYP2D6) involved in metabolite formation. In addition, two unknown putative metabolites have been detected. In vitro metabolite findings were confirmed in blood sample from a patient treated with quetiapine. This is the first report using LC-HRMS/MS untargeted screening and molecular networking to explore in vitro drug metabolism. Our data provide new evidences of the interest of molecular networking in drug metabolism exploration and allow our in vitro model consistency assessment.

## Introduction

Quetiapine is an orally administered atypical antipsychotic indicated in schizophrenia treatment, bipolar disorders and as an adjuvant treatment in major depressive disorders^1^. Widely prescribed in these indications, this dibenzodiazepine derivative shows affinity for various neurotransmitter receptors including serotonin, dopamine, histamine and adrenergic receptors and has binding characteristics at the dopamine-2 receptor similar to those of clozapine^2^. Quetiapine undergoes an extensive liver biotransformation, involving cytochromes P450 (CYPs) and uridine 5′-diphospho-glucuronosyltransferases (UGTs)^3–6^. Among them, CYP3A4 and CYP2D6 are the predominant metabolic systems : CYP3A4 is known to give rise to *N*-desalkylquetiapine, *N*-desalkylquetiapine sulfoxide and quetiapine sulfoxide and CYP2D6 is known to give rise to 7-hydroxyquetiapine, 7-hydroxy-*N*-desalkylquetiapine^3,6,7^. In addition, minor metabolism though CYP3A5 is known to give rise to *O*-desalkylquetiapine^7^. This knowledge on quetiapine metabolism is resulting from a sequence of works specifically targeting the metabolism of one molecule after another. Despite the recent design of appropriate bioinformatics tools, visualization tools allowing metabolism exploration are still underused.

Molecular networking (MN) allows the organization and representation of untargeted tandem mass spectrometry (MS/MS) data in a graphical form^8^. Each node represents an ion and its associated fragmentation spectrum, the links between the nodes indicating similarities between spectra. By allowing to propagate structural information within the network and facilitating sample-to-sample comparison, the MN approach offers valuable insights into drug metabolism^9^. Thereby, a multi-matrix approach provides a semi-quantitative visualization of molecule repartition in different matrix samples. MN has already proven its interest in plant species profiling^10^, metabolomics^11^, the dereplication of naturally produced substances^12^, and in drug metabolism analysis for in vivo clinical or forensic purposes^9,13^. Particularly, previous works focused on in vivo new psychoactive substances metabolite discovery^9^, multi-matrix post-mortem samples in case of drug intoxication^13^, and toxic plant samples in case of intoxication^14^. However, to our knowlegde, MN applied to in vitro metabolism exploration has not already been reported.

Many human hepatic cell lines, including HepG2 and HuH7 have been used as pharmacological and toxicological models. However, the lack of relevant expression of metabolism proteins is a major shortcoming when using cell cultures, particularly in drug biotransformation studies^15,16^. HepaRG, originally isolated from a female patient suffering from hepatocellular carcinoma, is a bipotent cell line that can differentiate into either cholangiocyte- or hepatocyte-like cells in appropriate culture conditions^17^. Differentiated hepatocyte-like HepaRG transcribe liver-specific genes at high levels, closer to primary human hepatocytes (pHH) and human liver tissue than any other liver cell lines^18,19^. More precisely, HepaRG cells express most of the drug processing genes including major CYPs^20^ and UGTs^19,21^. HepaRG cell culture exhibit a long-term fonctional stability while pHH loose their differentiated phenotype and drug metabolism over time. Moreover, HepaRG cells have been successfully used in metabolomics^22^ and have shown greater metabolite production compared to human liver microsome due to the lack of sulfation, methylation, acetylation or glutathione conjugation in this latter model^23^. Thus, there is a large body of evidence that differentiated HepaRG culture is a particularly relevant cell model system for drug metabolism study.

In the present study, MN application to in vitro metabolism exploration was investigated using our original MN analytical approach in differentiated HepaRG cells. Since in vivo quetiapine metabolism pathways generate well defined metabolite derivatives, we used this drug in order to explore the consistency of our in vitro metabolic model.

## Results

### In vitro quetiapine metabolism kinetic

**Figure 1:**
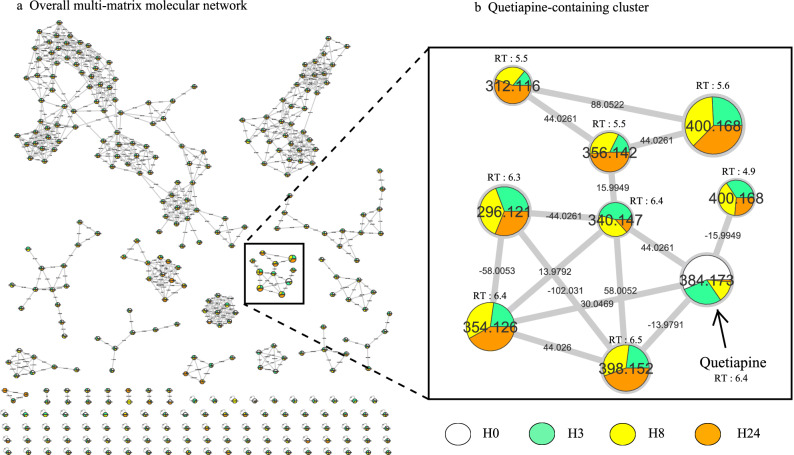
Visualization of in vitro quetiapine metabolism kinetics using molecular networking. Differentiated HepaRG were incubated with quetiapine (13 µM) during different incubation time. (**a**) The multi-matrix molecular network. Each incubation time is depicted in a specific color: H0 in white, H3 in green, H8 in yellow and H24 in orange. (**b**) Details of the specific quetiapine-containing cluster. Nodes are labelled with the exact protonated mass (*m/z*) and retention times (RT in minutes) and the links are labelled with the exact mass shift.

In order to understand quetiapine metabolism kinetic, we incubated quetiapine (13 µM) in metabolically competent human liver cell models, differentiated HepaRG cells during 0, 3, 8 and 24 h (H0, H3, H8 and H24, respectively). Analysis of culture media at different time allowed us to generate a multi-matrix molecular network which displayed the MS/MS data acquired during analysis (Fig. 1a). Nodes are labelled with the exact protonated mass (*m/z*) and retention times (RT in minutes) and the links are labelled with the exact mass shift. A specific color was assigned to each time (H0 in white, H3 in green, H8 in yellow and H24 in orange). The area of different color in each node represent concentrations of the corresponding compound in each condition in a semi-quantitative manner. Nodes were linked together in cluster according to their MS^2^ spectral similarities (Fig. 1a). Visual analysis of the multi-matrix molecular network shows a cluster containing quetiapine linked to other nodes (Fig. 1b). The molecular network allows to make two observations. First, quetiapine amount decrease as a function of incubation time with the HepaRG cells. Second, quetiapine is correlated with eight structurally related molecules (*m/z* 296.121 (RT: 6.3 min); 312.116 (RT: 5.5 min); 340.147 (RT: 6.4 min); 354.126 (RT: 6.4 min); 356.142 (RT: 5.5 min); 398.152 (RT: 6.5 min); 400.168 (RT: 4.9 min); 400.168 (RT: 5.6 min)). Immediately after quetiapine incubation (H0), none of these latter molecules was detectable. Taken together, these results suggest that these spectrally related molecules are putative quetiapine metabolites.

In this quetiapine-containing cluster (Fig. 1b), the related metabolites can be classified in two subgroups. The first subgroup corresponds to molecules that accumulate over time and therefore present their highest concentration at H24 (*m/z* 312.116; 354.126; 356.142; 398.152). The second subgroup corresponds to molecules that does not accumulate and present relatively constant concentrations over time as long as quetiapine remains [*m/z* 296.121; 340.147; 400.168 (RT: 4.9 min); 400.168 (RT: 5.6 min)] (Fig. 1b). Taken together, these visual results give a good understanding of quetiapine metabolism kinetic, and suggest that the quetiapine structurally closed molecules can be divided into metabolite accumulating over time and transitory metabolite which do not accumulate.

### Identification step

**Figure 2:**
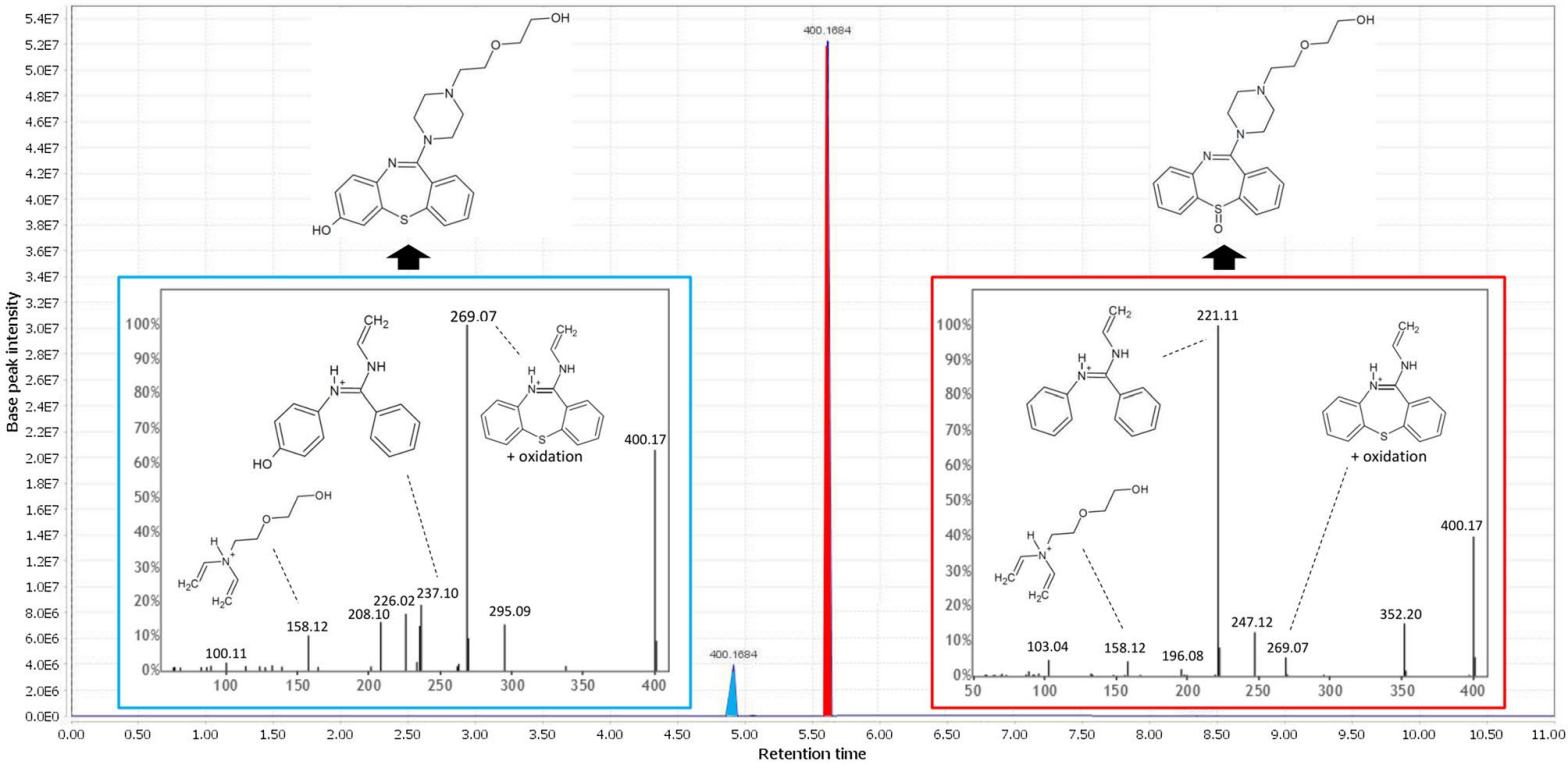
Isomer differentiation using. Chromatogram analysis of *m/z* 400.168 isomers (RT 4.9 and RT 5.6) at H24 and analysis of MS^2^ spectra allowed isomer differentiation. (**a**) Blue box: 7-hydroxyquetiapine MS^2^ spectra shows the oxidized cycle structure without sulfur fragment (*m/z* 237.10). (**b**) Red box: quetiapine sulfoxide MS^2^ spectra shows the unoxidized cycle without sulfur fragment (*m/z* 221.11).

Structural identification remains a challenge in metabolism exploration. Here, a node identified using GNPS or open spectral library can serve as a starting point to identify another node in the same cluster using information propagation. Information propagation within a molecular network consists in determining the structure of an unknown molecule using the structural information of the neighboring nodes. Such process can be automatized^24^. In particular, spectrally related molecules may display mass shifts corresponding to well-established biotransformation reactions (Supplementary Table 1)^13^. From a known structure, it is thus possible to determine the structure of a linked molecule, using these biotransformation reactions, coupled with structural information of the neighboring nodes. Our data showed also two compounds with the same exact mass (*m/z* 400.168) but with two different retention time (RT: 4.9 and 5.6 min) (Fig. 1b). According to literature data, *m/z* 400.168 could correspond to 7-hydroxyquetiapine (*m/z* 400.168) or quetiapine sulfoxide (*m/z* 400.168))^6^. These nodes are linked to quetiapine (*m/z* 384.173) with a mass shift of + 15.994, corresponding to oxidation reaction. We found also mass shifts which could correspond to two desalkylation (-44.026 Da), giving rise to *m/z* 296.121 compound (Fig. 1b). In order to find where the biotransformation took place, we used MZMine software chromatogram profile analysis and MS^2^ fragmentation trees analysis using SIRIUS 4.0.1 software^25^. Briefly, chromatogram profile analysis allows us to ensure that all nodes contained in the cluster of interest are not artefact. SIRIUS 4.0.1 software provides us a molecular formula for each detected fragment, allowing us to orientate towards a structural formula. Figure 2 displays the example of two *m/z* 400.168 isomers (RT 4.9 and 5.6 min).

We found that both isomers presented *m/z* 158.12 and *m/z* 269.07 fragments, which could correspond to the same alkyl chain of quetiapine and an oxidized tricycle, respectively. Thus, we concluded that oxidation reaction took place on the quetiapine tricycle. In addition, we found that *m/z* 237.10 and *m/z* 221.10 fragments correspond to an oxidized tricycle without sulfur and an unoxidized tricycle without sulfur, respectively (Fig. 2). These results suggest that *m/z* 400.168 (RT: 4.9 min) and *m/z* 400.168 (RT: 5.6 min) correspond to 7-OH-quetiapine and quetiapine sulfoxide, respectively. Same methodology was applied to other nodes, allowing identification of all the quetiapine-containing cluster molecules shown in Fig. 1.

### Quetiapine metabolic pathways exploration

**Figure 3:**
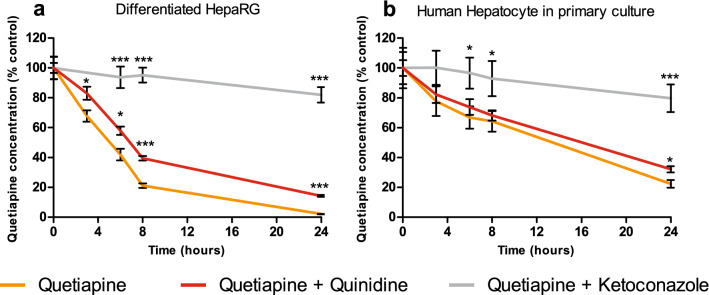
Cytochrome inhibitors enable quetiapine metabolic pathway inhibition in differentiated HepaRG and primary Human hepatocytes (pHH). Cells were incubated with quetiapine (13 µM) with or without CYPs inhibitors (ketoconazole 10 µM as a CYP3A4/5 inhibitor in grey; quinidine 10 µM as a CYP2D6 inhibitor in red) in (**a**) differentiated HepaRG cells or (**b**) pHH. Quetiapine concentration was measured at H0, H3, H6, H8 and H24 using high-resolution liquid-chromatography and expressed relative to the value determined at H0 (set arbitrarily to 100%). The data are quoted as the mean ± SEM from one experiment performed in triplicate. Statistics (unpaired student’s t-test): ***p < 0.01 and *p < 0.05 for cultures exposed to quetiapine + CYP inhibitor (ketoconazole or quinidine) compared with quetiapine alone at different times.

In order to explore metabolic pathways, we incubated quetiapine (13 µM) with or without cytochrome P450 (CYP) inhibitors during H24. Since Kittler et al. (2014) and Ferron et al. (2016) showed that ketoconazole was able to abolish CYP3A4-dependant metabolism, we adapted this method to our work^26,27^. Ketoconazole (10 µM) and quinidine (10 µM) were used as CYP3A4/5 and CYP2D6 inhibitors, respectively. Quetiapine quantitative analysis using a LC-HR-MS with external standard calibration curve method allowed us to control inhibitors effectiveness and measure quetiapine concentration at different time (Fig. 3). It also allowed us to compare these validated quantitative results with the semi-quantitative results obtained by MN. The relevance of HepaRG model (Fig. 3a) was assessed by comparing it to pHH in the same conditions (Fig. 3b). We observed a higher quetiapine metabolism in differentiated HepaRG cells compared to pHH. Indeed, approximately 50% of quetiapine were metabolized at H6 compared to 25% in pHH. However, of CYPs inhibitors displayed a similar profile between these two models. The use of quinidine showed a weak inhibition of quetiapine metabolism while ketoconazole allowed a strong inhibition. Taking together, these results suggest that (i) CYP3A4/5 is more likely involved than CYP2D6 in quetiapine metabolism and that (ii) differentiated HepaRG is a relevant model for this study.

**Figure 4:**
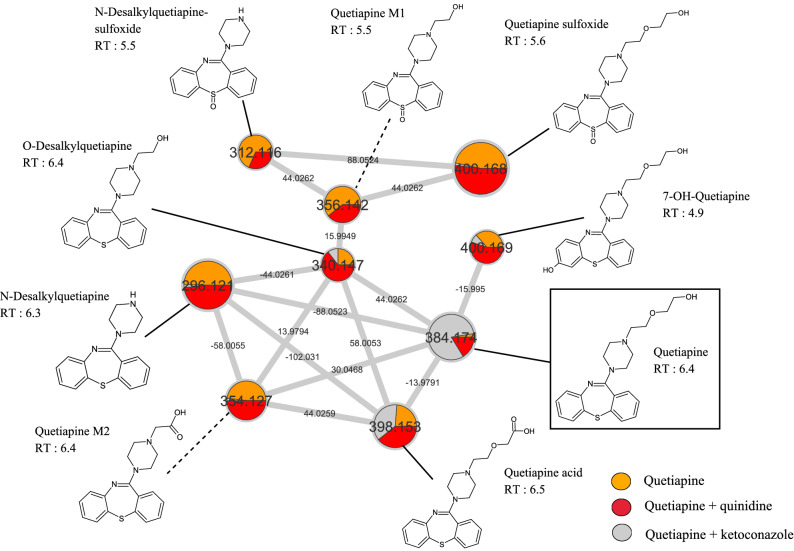
Extinction of CYP3A4/5 and CYP2D6 metabolic pathway visualization in differentiated HepaRG cells using molecular networking. Differentiated HepaRG were incubated with quetiapine (13 µM) during H24 with or without cytochrome inhibitors. Ketoconazole (10 µM) is used as a CYP3A4 inhibitor and quinidine (10 µM) is used as a CYP2D6 inhibitor. Each experimental condition is depicted in a specific color: quetiapine in orange, quetiapine + quinidine in red and quetiapine + ketoconazole in grey. Nodes are labelled with the exact protonated mass (*m/z*), chemical structures and retention times (RT in minutes) and the links are labelled with the exact mass shift.

We performed a MN approach in the presence of ketoconazole or quinidine CYP inhibitors in differentiated HepaRG cells to visualize what putative metabolites are CYP3A4/5- and/or CYP2D6-dependent. Since the aim was to compare metabolite accumulation with or without CYP inhibitor, the H24 time was chosen (Fig. 4). All the known molecules were named consistently with the literature data, conversely with unknown putative metabolites, here called quetiapine M1 (*m/z* 356.142; RT: 5.5 min) and quetiapine M2 (*m/z* 354.127; RT: 6.4 min).

We observed that quetiapine (*m/z* 382.174) incubated alone (in orange) is nearly absent of culture media at H24 in Fig. 4. In addition, quetiapine incubated with quinidine (in red) showed a lower amount at H24, compared with quetiapine incubated with ketoconazole (in grey). This corroborate our quantitative results, showing a minor metabolism through CYP2D6 (Fig. 3). We found that a few putative metabolites were nearly absent of culture media when incubated with ketoconazole (in grey), suggesting a CYP3A4/5-dependent metabolic pathway for (N-desalkyquetiapine, Quetiapine M2, 7-OH-quetiapine and quetiapine sulfoxide). Similarly, production of some putative metabolites was decreased in the presence of quinidine (in red), suggesting a CYP2D6-dependent metabolic pathway (N-desalkyquetiapine sulfoxide and quetiapine M1). We also observe that a few metabolites undergo metabolism through these two isoenzymes (quetiapine M1, *N*-desalkylquetiapine sulfoxide and *O*-desalkylquetiapine). The same experiment was carried out in our pHH model (Supplementary Fig. 1). However, fewer metabolites were found, which is consistent with our quantitative results showing a slower metabolism in this model (Fig. 3). Taken together, these results show that MN allows detailed visual analysis of in vitro metabolism, including metabolic pathways.

### In vivo versus in vitro quetiapine metabolism

**Figure 5:**
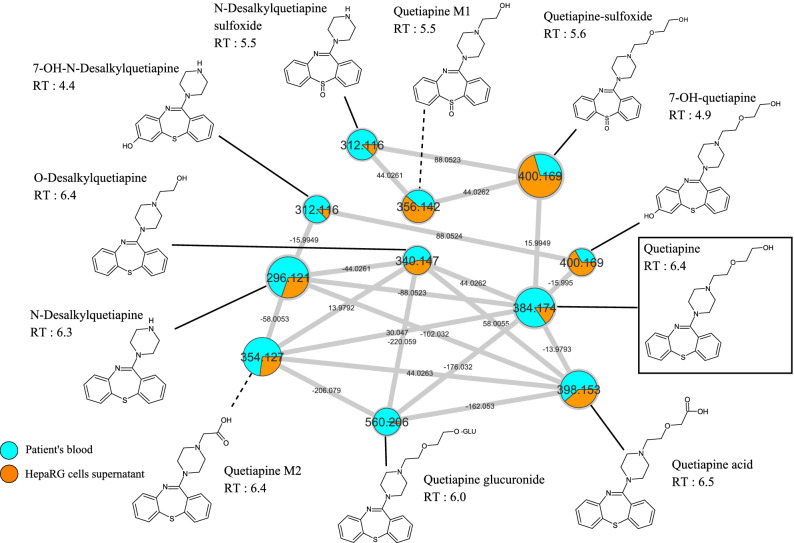
in vivo versus in vitro quetiapine metabolism. Blood sample from a patient treated with quetiapine was compared with differentiated HepaRG cells culture media incubated with quetiapine (13 µM) during H24. In this quetiapine-containing cluster, patient sample and differentiated HepaRG cells supernatant are depicted in blue and orange, respectively. Nodes are labelled with the exact protonated mass (*m/z*), chemical structures, and retention times and the links are labelled with the exact mass shift.

In order to compare our in vitro findings to in vivo data, we performed a sample-to-sample comparison using MN, including one blood sample of patient treated by quetiapine and our culture media of differentiated HepaRG incubated by quetiapine (13 µM) during H24 (Fig. 5).

Quetiapine-containing cluster visual analysis revealed that all putative metabolites found in our previous in vitro experiment were present in the patient blood sample (N-desalkylquetiapine, N-desalkyquetiapine sulfoxide, quetiapine M1, quetiapine M2, O-desalkylquetiapine, 7-OH-quetiapine and quetiapine sulfoxide and quetiapine acid). In addition, we found two other metabolites, present in both blood sample and differentiated HepaRG cells supernatant, resulting from oxidation of N-desalkylquetiapine (*m/z* 312.116; RT: 4.4) and glucuronidation (+ 176.032) of quetiapine (*m/z* 560.206). Identification step suggested that these two metabolites could correspond to 7-OH-*N*-desalkylquetiapine and quetiapine glucuronide, respectively. Taken together, these results suggest that differentiated HepaRG is a strong and consistent model in metabolism study. Sample-to-sample comparison including in vivo samples seems to be is a potent tool to further explore in vitro data experiment. Supplementary Table 2 reports putative identified compounds or metabolites contained in the quetiapine-containing clusters, and metabolic pathway involved in quetiapine metabolites biotransformation.

## Discussion

Metabolism unraveling is a fundamental objective of pharmacologic studies. However, visualization tools allowing quick metabolism exploration are still under-used. The aim of this study is to illustrate the interest of molecular network for in vitro drug metabolism exploration.

Quetiapine appeared as a suitable candidate drug to carry out this proof of concept. Indeed, this molecule undergoes an extensive and well-described metabolism. The existence of an abundant literature allows us to compare it with our data in order to assess their relevance^3–7^. In addition, it is an inexpensive and widely prescribed molecule. Samples from patients treated with quetiapine were then easily available to compare real-life data with our in vitro study.

Identification means were also of major importance in this study. For this purpose, we used validated process based on exact mass, mass spectra, retention times, fragmentation pattern analysis with SIRIUS 4.0.1 software, information propagation, and mass shifts corresponding to biotransformation reactions as already described^9,13,25^. Here, we propose identification of 10 putative quetiapine metabolites, among which two of them (quetiapine M1 and M2) have not been already described in the literature (Supplementary Table 2). Kinetic metabolism study brings us valuable data on quetiapine-related nodes. Indeed, by visualizing quetiapine clearance, correlated with structurally related molecules appearance, we present strong evidence for claiming they are metabolites. None of them was detectable before H3 incubation and since their intermediate and final metabolite profils could be distinguished, a good understanding of the metabolites filiation can be proposed. These data allowed us to bring additional evidence on quetiapine metabolic pathway (Supplementary Fig. 2).

To date, it is well established that quetiapine is primarily metabolized by CYP3A4, CYP2D6, and CYP3A5 to a lesser extent. CYP3A5 appears to be routinely present in human kidney tissue but it is detected in only 25 to 30% of adult liver samples^28,29^. Thus, pHH model may be limited by the lack of this cytochrome expression in a donor. This limitation was partially overcome using differentiated HepaRG cells that exhibit poor but present metabolizer alleles for CYP3A5^30^. To further investigate quetiapine metabolism pathways, we used quetiapine metabolism inhibitors. Ketoconazole is well known to be a CYP3A inhibitor, including CYP3A4 and CYP3A5, having high inhibitory potency and producing reversible inhibition through competitive and non-competitive mechanisms^31–37^. In this way, in vitro studies have shown that 10 µM ketoconazole inhibits specific activity of CYP3A4 on HepaRG cells^27^. Since ketoconazole is known to inhibit CYP3A4 and CYP3A5, we were not able to precisely determine which cytochrome was the most involved in metabolites production. Therefore, we chose to mention CYPA4/5 in Supplementary Fig. 2 and Supplementary Table 2. In addition, a potent competitive inhibitor of CYP2D6 is quinidine^38–41^. For these reasons, these two molecules (ketoconazole and quinidine) have been used in this study to clarify metabolism pathways. Metabolism inhibition visualization using MN brings further insight into metabolism pathways mapping and allows us to objectify a predominant CYP3A4/5 metabolism.

Comparing HepaRG data with in vivo data appears critical to establish the proof of concept concerning the use of molecular network in in vitro drug metabolism exploration. Among the 10 known quetiapine metabolites (Supplementary Fig. 2)^3,5–7^, we identify 8 of them (*N*-desalkylquetiapine, *N*-desalkyquetiapine sulfoxide, 7-OH-*N*-desalkyquetiapine, *O*-desalkylquetiapine, 7-OH-quetiapine, quetiapine sulfoxide, quetiapine acid and quetiapine glucuronide) whereas two other compounds were unknown putatives metabolites (quetiapine M1 and quetiapine M2). The 10 putative metabolites reported in this study have been found in both human sample and HepaRG cells supernatant, showing the consistency of this latter model. Furthermore, by comparing in vitro and in vivo data, we found two metabolites that were not seen in previous in vitro molecular networks (quetiapine glucuronide and 7-OH-*N*-desalkylquetiapine). Since these latter metabolites were found to be equally present in in vitro experiments according to exact mass and retention time comparison at the gap-filling step, we believe that this preprocess made it possible to link the information of weakly expressed molecules in vitro. Thus, sample-to-sample comparison is found to be a potent tool to visualize weakly expressed molecules.

However, as expected, MN must be interpreted considering several limitations and requires optimizations at every stage of data processing, and a good knowledge of LC–MS/MS analysis. We cannot exclude that different LC–MS/MS settings would have allowed the visualization of other metabolites, in particular phase II metabolites.

We demonstrated that organizing data by their spectral similarities is a useful means to identifying new metabolites. Furthermore, unknown drug metabolite identification can provide valuable information on drug toxicity. It would be interesting to consider broader perspectives on metabolite mapping in the context of toxicity studies^42^. in vitro cell line models such as HepaRG can be used to study pathophysiology mechanism of diseases like steatosis and NAFLD^43^. Moreover, next-generation liver organoids can be produced directly from patient’s cells (ie blood cells, urine cells) and modified genetically to study patient diseases and regenerative medicine purposes^44^. Those tools combined with MN approaches provide a new way to integrate patient individual variability in therapeutic efficiency and toxicity studies.

## Material and methods

### Material

William’s E medium (ref: 12551032) was purchased from Gibco (ThermoFischer Scientific, San Jose, CA). Penicillin–streptomycin was obtained from Life Technologies (Grand Island, NY USA). Fetal Bovine Serum (FBS) was purchased from Eurobio (Courtaboeuf, France) and from Hyclone GE Healthcare Life Sciences (Logan, UT USA). Hydrocortisone hemisuccinate was purchased from Serb (Paris, France). Dimethyl sulfoxide (DMSO), formic acid, insulin, ketoconazole and quinidine were obtained from Sigma-Aldrich (Saint Louis, MO USA). Quetiapine was purchased from LGC Standards (Teddington, UK).

### Cell culture and treatment

Progenitor HepaRG cells were cultured as previously described^20^. Briefly, HepaRG cells were seeded at a density of 10^5^ cells/well in 96-well plates and cultured during two weeks in culture medium (William’s E medium (1X) (A12176-01, Gibco) supplemented with 10% FBS, 50 U/mL penicillin, 50 μg/mL streptomycin, 5 µg/mL insulin, 2 mM glutamine, 50 μM sodium hydrocortisone hemisuccinate and 2% DMSO). Cells were then cultured during two more weeks in the same medium supplemented with 2% DMSO to induce cell differentiation into cholangiocyte- and hepatocyte like cells^17^. The detection of quetiapine and its metabolites was performed using this coculture model. pHH were obtained from the processing of biological samples through the Centre de Ressources Biologiques (CRB) Santé of Rennes BB-0033-00,056 under French legal guidelines and fulfilled the requirements of the institutional ethics committee. Cells were isolated by collagenase-perfusion of liver biopsies from adult donors^45^ and these cells were plated in 96-well plate at a density of 1.5 × 10^5^ /cm^2^ and cultured in the same William’s E medium than HepaRG cells supplemented with 2% of DMSO. Cells were cultivated 4 days after plating prior to compounds exposure.

Differentiated HepaRG cells and pHH were incubated with 100 µL of quetiapine (13 µM) during H0, H3, H6, H8 and H24 with or without CYP inhibitors during all the treatment time. Ketoconazole (10 µM) and quinidine (10 µM) were chosen in order to inhibit CYP3A4 and CYP2D6, respectively.

### Samples extraction

in vitro samples (25 µL) and in vivo blood samples (200 µL) obtained from one patient at the Toxicology Laboratory of Rennes University Hospital were extracted as already described^13,14^. Briefly, samples were supplemented with 500 µL of methanol containing internal standard (risperidone-D4) and then extracted with 300 μL of 0.1 M zinc sulfate solution. After supernatant evaporation, the residue was dissolved in 200 μL of LC–MS grade water and transferred into chromatographic vials for LC-HR-MS analysis and quantification. In order to extract and analyze samples using non-targeted screening, 25 µL of each sample were supplemented with 75 µL of methanol. The supernatant (50 µL) was then diluted with LC–MS grade water (50 µL) and transferred into chromatographic vials for LC-HR-MS analysis.

### LC–MS settings

Liquid chromatography-mass spectrometry (LC–MS) analyses were carried out using Orbitrap Q Exactive mass spectrometer coupled to an UltiMate 3000 pump (Thermo Scientific, San Jose, CA). A heated electrospray ionization source (HESI-II) was used for the ionization of the target compounds. Data acquisition, calibration and instrument control were performed using Xcalibur 2.1 (Thermo Scientific, San Jose, CA) software. Samples were maintained at 15 °C in the autosampler and quality controls were injected before each analysis.

Quetiapine quantitation assays were performed using a validated LC-HRMS method as already used^13^ as follow: The mobile phases were composed of ammonium formiate at 10 mM and formic acid 0.1% in water (phase A) and acetonitrile and formic acid 0.1% phase B). LC was performed on a on a Hypersil Gold column (5 mm × 2.1 mm, 3 µm) (Thermo Scientific, San Jose, CA).

Gradient elution was as follow : initial conditions of 95:5 (A:B) maintained for 1.5 min, increasing to 70:30 (A:B) for 3.5 min, increasing to 60:40 (A:B) for 3 min, increasing to 5:95 (A:B) for 1 min, followed by a 1 min plateau with 5:95 (A:B), decreasing to 95:5 (A:B) for 3 min, and return to initial conditions 95:5 (A:B) for equilibration for 3 min. This corresponded to a total chromatographic run of 15 min. The flow rate was 500 μL/min, the column temperature was maintained at 25 °C, the injection volume was 5 μL. For mass spectrometry, the instrument operated in ESI positive mode, the range for acquisition was 120–700 m*/z*. Full scan data were acquired at a resolution of 140.000 FWHM, with an AGC target of 1e6 and a maximum injection time of 200 ms. Source parameters were as follows: source voltage + 4.5 kV, sheath gas flow 35 units, auxiliary gas flow 15 units, capillary temperature 300 °C, S-Lens RF level 50 units.

Non-targeted screening LC-HRMS/MS method used for MN building was as follow: The mobile phases were composed of ammonium formiate at 2 mM and formic acid 0.1% in water (phase A) and ammonium formiate at 2 mM and formic acid 0.1% in methanol and acetonitrile (50/50) (phase B). LC was performed on a Accucore Phenyl Hexyl (100 × 2.1 mm, 2.6 μm) (Thermo Scientific, San Jose, CA) using the following gradient elution: initial conditions of 99:1 (A:B) maintained for 1 min, increasing to 1:99 (A:B) for 9 min, followed by a 1.5 min plateau with 1:99 (A:B) and return to initial conditions 99:1 (A:B) for equilibration. This corresponded to a total chromatographic run of 15 min. The flow rate was 500 μL/min, the column temperature was maintained at 40 °C, the injection volume was 10 μL. For mass spectrometry, the instrument operated alternately in ESI positive and negative mode in the same run, the range for acquisition was, respectively, 70–1000 m/z in positive mode and negative mode. Ion precursor selection was performed in the data dependent mode of operation where the most intense ion from the previous scan was selected for fragmentation. Full scan (MS1) data were acquired for each ionization mode at a resolution of 35,000 FWHM, with an AGC target of 1e6 and a maximum injection time of 120 ms. Source parameters were as follows: source voltage + 3.0 and − 4.0 kV, sheath gas flow 60 units, auxiliary gas flow 10 units, capillary temperature 320 °C, S-Lens RF level 60 units. MS/MS (MS2) data were acquired at a resolution of 17,500 FWHM with an AGC target of 1e5, maximum injection time was 50 ms, a TopN of 5 in positive mode and 2 in negative mode, an isolation window of 2.0 m/z. The normalized collision energy (NCE) was stepped at 17.5, 35 and 52.5, and the dynamic exclusion time set at 3 s.

### MN generation

Spectral data allowed us to generate MN using semi-quantitative bioinformatics approach. Data acquisition, processing (i.e. MS data conversion, preprocessing, MS1 annotation, and generation of molecular networks), visualization and network analysis have been described in detail elsewhere^9^. Briefly, raw data were converted to an open MS format (.mzXML) with ProteoWizard’s MSConvert module^46^. The mzXML files were then preprocessed (deconvolution, de-isotoping, alignment, gap-filling) with MZmine 2 software^47^. The single .mgf output file was then loaded on the Global Natural Products Social networking (GNPS) web-based platform in order to generate the multi-matrix molecular network^8^. To the use of high resolution data, the basic parameters were modified to *m/z* 0.02 for the mass tolerance of precursor and fragment ions used for MS/MS spectral library searching, and *m/z* 0.02 for the mass tolerance of fragment ions used for MN. The minimum cluster size was set to 1. In addition, links between nodes were created when the cosine score was greater than 0.70, and the minimum number of common fragment ions shared by two MS/MS spectra was 6. Links between two nodes were only kept in the network if each node was in the top 10 most similar nodes. Full data processed through the GNPS platform are accessible through these links:

- https://gnps.ucsd.edu/ProteoSAFe/status.jsp?task=028beffa249c495dbe8c9c94dfbc3e49 (Fig. 1: in vitro quetiapine metabolism kinetic);
- https://gnps.ucsd.edu/ProteoSAFe/status.jsp?task=cfabffd4f1cf4fbb8571bb039040ab75 (Fig. 4: in vitro quetiapine metabolism pathway inhibition in differentiated HepaRG cells);
- https://gnps.ucsd.edu/ProteoSAFe/status.jsp?task=dfe75ac4273d49b69b832bbe3c2c5ef5 (Fig. 5: in vitro versus in vivo quetiapine metabolism);
- https://gnps.ucsd.edu/ProteoSAFe/status.jsp?task=8f00f95fd3ca4ea99fc6477ba1a55caf (Supplementary Fig. 1: in vitro quetiapine metabolism pathway inhibition in pHH).

The molecular network was visualized using Cytoscape 3.5.1 software^48^. The nodes were annotated by comparison with reference standards, by spectral matching with the curated GNPS, mzCloud online mass spectral libraries and information propagation^49^.

## Supplementary information

Supplementary information.
