# Supplementary material for: New insights into quetiapine metabolism using molecular networking
Source: Sci Rep. 2020 Nov 16;10:19921. doi: 10.1038/s41598-020-77106-x (PMC7669884; doi:10.1038/s41598-020-77106-x)
Supplement: Supplementary file 1 — Supplementary information. [file 41598_2020_77106_MOESM1_ESM.pdf]

New insights into quetiapine metabolism using molecular networking

Brendan Le Daré[1,2*], Pierre-Jean Ferron[1], Pierre-Marie Allard[3], Bruno Clément[1], Isabelle Morel[1,2], Thomas Gicquel[1,2]

1. *Univ Rennes, INSERM, INRAE, CHU Rennes, Institut NuMeCan (Nutrition, Metabolism and Cancer), PREVITOX network, F-35033 Rennes, France.*
2. *Rennes University Hospital, Forensic Toxicology Laboratory, F-35033 Rennes, France.*
3. *School of Pharmaceutical Sciences, and Institute of Pharmaceutical Sciences of Western Switzerland (ISPSW), University of Geneva, CMU, Rue Michel Servet 1, 1211 Geneva 4, Switzerland*

Corresponding author: Dr. Brendan Le Daré (ORCID**:** 0000-0002-5907-2450 and Publons: AAL-7050-2020)

Address : Laboratoire de toxicologie biologique et medico-légale, CHU Pontchaillou, 2 Rue Henri Le Guilloux, 35000 Rennes.

E-mail: brendan.le.dare@chu-rennes.fr

SUPLLEMENTARY DATA

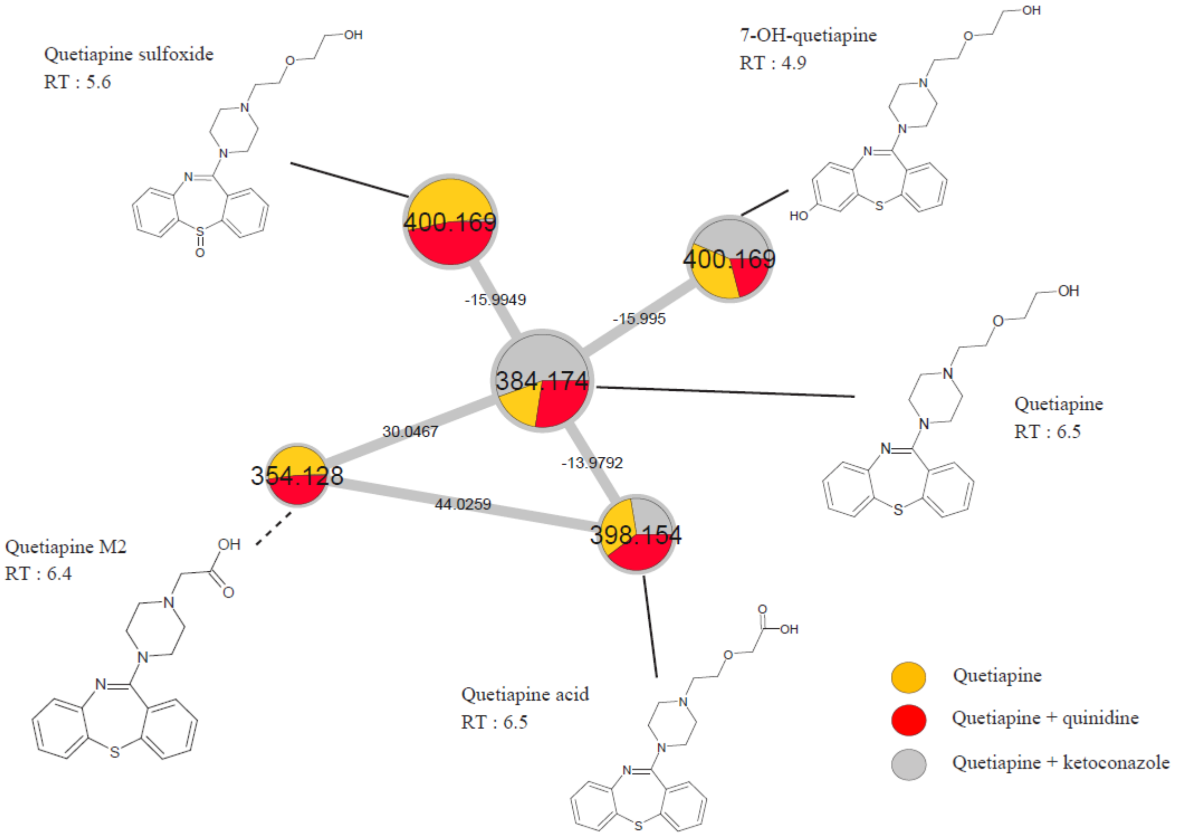

Supplementary Figure 1: Extinction of CYP3A4/5 and CYP2D6 metabolic pathway in primary Human hepatocytes (pHH) using molecular networking. pHH were incubated with quetiapine (13 µM) during H24 with or without cytochrome inhibitors. Ketoconazole (10 µM) is used as a CYP3A4/5 inhibitor and quinidine (10µM) is used as a CYP2D6 inhibitor. Each experimental condition is depicted in a specific color: quetiapine in white, quetiapine + quinidine in red, quetiapine + ketoconazole in grey and quetiapine + quinidine + ketoconazole in green. Nodes are labelled with the exact protonated mass (*m/z*), chemical structures and the links are labelled with the exact mass shift.

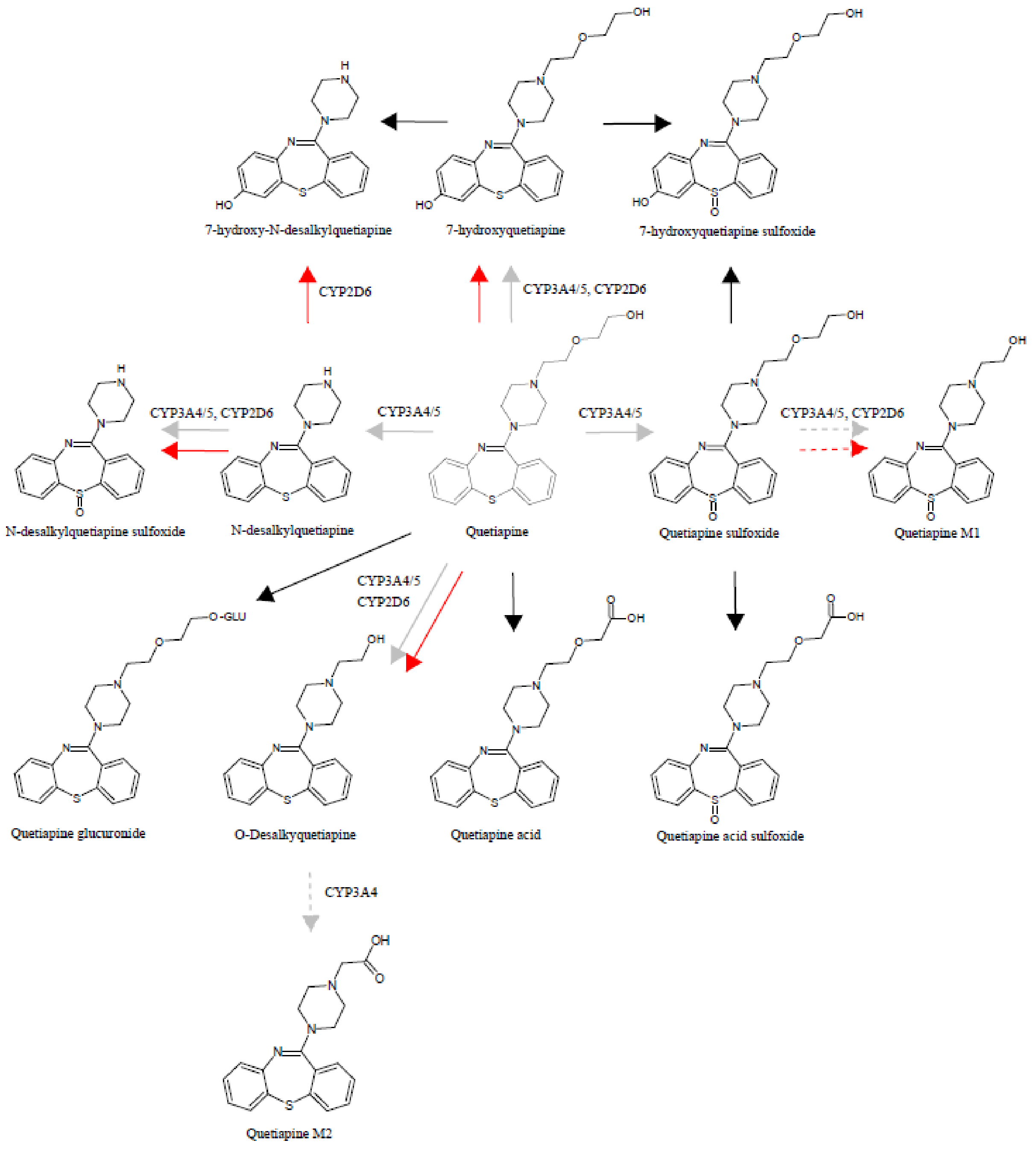

Supplementary Figure 2: Quetiapine metabolism overview. New putative identified metabolites are shown with a dash line. Metabolites for which metabolism involve CYP3A4/5 or CYP2D6 are shown with grey and red arrow, respectively.

Supplementary Table 1: Exact mass shift differences corresponding to biotransformations reactions

**Biotransformation**	**Corresponding formula**	**Exact mass shift (Da)**
Oxidation + Dehydrogenation	+ H2 -O	13.970
Oxidation	+ O	15.994
Deacetylation + Dehydrogenation	- C2H4O	44.026
Acetylation + oxidation	+ C2H2O2	58.005
Glucuronidation	+ C6H8O6	176.032

Supplementary Table 2 Putative identified compounds or metabolites contained in the molecular network.

t_R (min)	Observed *m/z* [M+H$^+$]	Theorical *m/z* [M+H$^+$]	Molecular formula	Δ exact mass error	ESI-HRMS² data (*m/z*)	Putative compound identification	CYP involved in compound production and/or metabolism
4.4	312.1168	312.1170	C17H17N3SO	0.0002	70.07; 84.96; 89.06; 103.04; 226.03; 243.06; 269.07; 297.15	7-hydroxy-N-desalkylquetiapine	-
4.9	400.1691	400.1695	C21H25N3O3S	0.0004	70.07; 96.08; 158.12; 208.10; 226.03; 237.10; 263.12; 269.07; 295.09	7-hydroxyquetiapine	CYP3A4/5
5.5	356.1428	356.1432	C19H21N3O2S	0.0004	90.98; 103.04; 114.09; 221.11; 247.12; 269.07; 308.18	Quetiapine M1	CYP3A4/5, CYP2D6
5.5	312.1162	312.1170	C17H17N3SO	0.0008	70.07; 195.09; 221.11; 247.12; 264.15	N-desalkylquetiapine sulfoxide	CYP3A4/5, CYP2D6
5.6	400.1690	400.1695	C21H25N3O3S	0.0005	68.05; 70.07; 158.12; 196.08; 221.11; 247.12; 269.07; 352.20;	Quetiapine sulfoxide	CYP3A4/5
6.0	560.2066	560.2066	C27H33N3O8S	0.0000	70.07; 96.08; 132.10; 158.12; 210.04; 221.11; 247.12; 253.08; 279.10; 384.17	Quetiapine glucuronide	None
6.3	296.1217	296.1221	C17H17N3S	0.0004	70.07; 210.04; 221.11; 227.06; 253.08; 279.10	N-desalkyquetiapine (norquetiapine)	CYP3A4/5
6.4	340.1478	340.1483	C19H21N3O1S	0.0005	88.08; 114.09; 210.04; 221.11; 247.12; 253.08; 279.10	O-desalkyquetiapine	CYP3A4/5, CYP2D6
6.4	354.1273	354.1276	C19H19N3O2S	0.0003	59.05; 84.08; 210.04; 221.11; 247.12; 253.08; 279.10	Quetiapine M2	CYP3A4/5
6.4	384.1741	384.1745	C21H25N3O2S	0.0004	59.05; 73.03; 158.12; 210.04; 221.11; 247.12; 253.08; 279.10	Quetiapine	CYP3A4/5, CYP2D6
6.5	398.1534	398.1538	C21H23N3O3S	0.0004	70.07; 103.04; 146.08; 172.10; 210.04; 221.11; 247.12; 253.08; 279.10	Quetiapine acid	?
